# A Treatise on the Etiology, Pathology and Treatment of Congenital Dislocations of the Head of the Femur

**Published:** 1850-05

**Authors:** 


### BIBLIOGRAPHICAL NOTICES.

*A Treatise on the Etiology, Pathology and Treatment of Congenital Dislocations of the Head of the Femur.* Illustrated with plates. By JOHN MURRAY CARNACHAN, M. D., Lecturer on Operative Surgery with Surgical and Pathological Anatomy, &c. &c. New York : S. S. & W. Wood, 261 Pearl street.

The progression which has taken place in the science of medicine within the last few years, cannot fail to prove a source of abiding interest to all engaged in its pursuit. Nothing, perhaps, demonstrates more clearly that its progress is advancing rapidly, not toward perfection and certainty, but towards a higher position than it has heretofore reached, than the fact that its different and special departments are assiduously cultivated by accurate and industrious observers, who are ready and willing to confer on their fellow-laborers the benefits of their experience and investigations. The field of science, like that of agriculture, may be most advantageously cultivated when judiciously divided, and especially when to every division some capable and accomplished laborer is willing to devote himself, and to contribute the fruits of his labors to the common stock of others concerned in its culture.

Special departments of medicine have long since been laid out, and the sub-division is now so extensive, that there is scarcely a subject of importance on which monographs have not appeared, which, while they reflect credit on the ability and research of their authors, prove to the profession of inestimable value.

The work before us, giving an account of a special department of surgery, cannot fail to prove of interest to the practical surgeon, as it appears to be an elaborate and faithful delineation of a very distressing and badly understood affection, which, until recently, has been deemed incurable, and to which the attention of the profession has been directed only within the last few years.

In the preface, our author dwells upon the necessity of an accurate knowledge of anatomy, physiology, and pathology, to the surgeon, and throughout the book he demonstrates not only his willingness, but also his ability, to follow up his precept by example, in the full description of congenital dislocations of the head of the femur upon the dorsum of the ilium, and by the details of his individual investigations as to the different conditions of the affection.

The work entitled "A Treatise on the Etiology, Pathology, and Treatment of Congenital Dislocations of the Head of the Femur," is divided into seven chapters, the whole occupying 235 pages, and illustrated by thirteen well executed engravings.

The 1st chapter is occupied with "General considerations on congenital dislocations of the head of the femur," and a history is given of most that has been published on the subject since the days of Hippocrates, who first noticed that children, while in utero, may be affected with various deformities, and among them with certain dislocations, without dwelling at all on the variety under consideration. In latter times, the affection has been studied by Ambrose Paré, Verdue, &c., who demonstrate the possibility of the occurrence of this and other dislocations, while the child is still in utero, which almost inevitably prove the source of deformity for life. Dislocations occurring, no matter at what period, or how, are of deep interest to the surgeon, as on his efforts alone can the misfortunes which would otherwise follow them be averted. Unhappily, from the difficulties of diagnosis attending those occurring congenitally, and the insufficiency of the means for their reduction, they are usually allowed to pass unnoticed, until certain changes have taken place in the structures about the articulation, which may forever preclude the possibility of a cure.

Our author quotes from Paré, Palleta, and Dupuytren, in proof that these malformations do take place, and that they occur much more frequently than is usually supposed, and also shows that these writers were aware of the difficulties to be encountered in the diagnosis of them, and especially between these and others, which are described as pseudo-luxations and sub-luxations. He gives a general description of the malformation, and of the changes which ultimately follow in the structures in the neighbourhood of the articulation.

In Chapter 2d, the anatomical relations of the joint, the bones composing it, with the ligaments, &c., are carefully described, and the difference between the articulation as perfected in the adult, and its imperfect or rudimentary state in the fœtus or even in the child for some time after birth, is given, and the facility with which the head of the femur may escape from the acetabulum, is shown, not only from the shallowness of the cavity, but also from the want of perfection in the conformation of the head of the bone itself.

The position of the child in utero, with the thighs strongly flexed upon the pelvis, is considered favorable to the escape of the head of the femur, whenever there is any irregularity in the development of the parts about the pelvi-femoral articulation, or whenever the muscles, already on the stretch, are brought into active contraction. The conditions of the articulation favorable to the accident, are very well shown in *plate No.* 3.

In Chapter 3d, the etiology of the deformity is very elaborately entered into, and although the author is decided in his views as to its production, he is still candid in giving the opinions of others, notwithstanding the weight of their authority.

A distinction is made between terms used indiscriminately, though of different meaning, as expressive of the affection, such as *spontaneous*, *original*, *congenital*, and the latter is adopted as most appropriate under all circumstances. Congenital luxations of the head of the femur, are divided (as when occurring after birth) into complete and incomplete, and the directions in which they may take place, are three, 1st, upwards and outwards; 2d, directly upwards, and 3d, forwards and upwards. The first is the variety which is of the most frequent occurrence, and which is most likely to take place from the natural relations of the parts; the two last are stated to occur as forms of fœtal monstrosities. A sub-luxation, described by M. Guérin, as occurring upwards and backwards, and two varieties of *pseudo-luxation*, of the same author, are given, though not dwelt upon; the first simulating a luxation backwards and outwards; the second, downwards and forwards; the head of the femur in neither instance having escaped from the acetabular cavity. One author considers these varieties as belonging to the imperfectly developed fœtus or to monsters, and leaves them with the intention of giving a more full account of those which more especially "belong to the practical details of the profession."

The causes given by different writers as producing the affection under consideration are—first. External violence acting upon the fœtus while in utero. (*J. L. Petit.*) Second. A primitive alteration in the germ, or an aberration of the formative power—(*force formatrice.*) (*Dupuytren.*) Third. An arrest in the development of the osseous portions forming the cotyloid cavity. (*Breschet.*) Fourth. Certain articular maladies occurring in the fœtus during intra-uterine life. (*Revived by M. Parise, and others.*) Fifth. A primitive alteration in the nervous centres. (*Chaussier*) revived by Delpech and Guérin; and lastly, to be more definite; I would add to the causes mentioned above, a pathological spasmodic retraction of the muscular tissue, *resulting from a perverted or disturbed condition of the excito-motor apparatus of the medulla spinosis; especially of that portion which is in direct relation with the nervous branches distributed among the pelvi-femoral muscles.*"

Discussing fully the first four causes as stated, our author discards them, believing the last, which, to a certain extent, embraces the fifth, enough to account for the production of the affection. He gives at length the causes which induce muscular contraction, and the aberrations from the normal conditions. Indeed, his remarks constitute a resumè of the doctrine of excito-motor function, and he bases on its derangement the production of congenital dislocations of the head of the femur, as well as of other deformities which doubtless have the same etiology. He combats freely the doctrine advanced by *Chaussier* and *Palleta*, and subsequently adopted by *Rudolphi*, *Delpeck*, and *Guérin*, of the absence of or alteration in any parts of the nervous centres, and argues to prove that the aberration may be and is entirely functional, which is certainly nothing more than reasonable, when we know that children are born with every variety of malformation, and yet both brain and spinal column are found to be in a perfectly healthy condition. The anatomical relations of the nervous system as bearing on the argument, are clearly pointed out, and many functional derangements given in support of what our author believes the true causes of these congenital dislocations, viz., *morbid, muscular, spasmodic contractions*, and to which he attributes the dislocation under consideration.

In Chapter 4th, the symptomatology of the affection is described, and the difference between the appearances presented at the different periods of life is considered.

Our author thinks that the affection may be entirely overlooked in the infant or young subject, but believes that when the attention is once directed to the articulation, the nature of the case becomes evident, as the appearances are very much the same as those presented in traumatic luxation of the femur, while the muscles in the neighborhood of the joint are usually so atrophied as to render a comprehension of the relation of the parts sufficiently easy.

The deformity, which may be slight at first, is increased so soon as the child supports itself, when the weight of the body is thrown upon the coxi-femoral articulation, inasmuch as the head of the femur, which at first rests just without the socket, is then forced higher up, until it ultimately assumes a position on the dorsum ilii. Strange to say, the dislocation of both bones is more common than that of one alone. When such is the case, the diagnosis is rendered more difficult, inasmuch as there is then no inequality in the length of the limbs. When such is the case, the disproportion between the length of the body and inferior extremities is striking; there is a curvature forwards of the lumbar region, the pubic region is tilted forwards and downwards, and the lower portion of the trunk appears to be sunken between the upper portion of the thighs. From this circumstance, the arms, when hanging by the sides, appear too long. The trochanters project abnormally, and the contour of the hips is altered; the fold in the groin is deeper than usual, and the extremities, from want of use, are not fully developed. The direction of the limbs varies, and the locomotion of the patient is difficult, and "a kind of double lameness is produced, somewhat resembling the hobbling motion of the duck." When the patient is placed in the recumbent posture, the limbs may be elongated, and many of the symptoms observable to the eye disappear. Among the appearances presented to the eye, as well as the signs manifested by manual manipulation, the absence of the head of the femur from its natural position when the limb is rotated, while the fingers are placed in the fold of the groin, is looked upon both by our author and M. Prevaz as "a certain sign of displacement."

The different measurements and the motions which may be given to the limbs, are well described, and the appearances presented are illustrated by a reference to the plates. The symptoms, when the head of one femur only is dislocated, are also well pointed out, and an abstract is given of a well written paper on congenital dislocations of the femur, published by our author in the London Lancet, in 1844, which seems to cover the whole ground.

Chapter 5th.—*Diagnosis.* The diseases with which this deformity may be comfounded are: morbus coxarius, sub-luxation of the femur, the pseudo-luxations of M. Guérin, malformations about the joint, osseous deposits, and shortening of the limbs. Our author thinks that the appearances given are sufficiently distinctive when carefully studied, and that the present symptoms and history of morbus coxarius will prevent any mistake so far as it is concerned.

Chapter 6th.—*Prognosis.* Notwithstanding the opinion of M. Dupuytren, as to the incurability of the affection, our author assumes its curability, and subsequently shows that cases have been successfully treated, when the patients have been young. He, however, cautions the surgeon against a too favorable prognosis. He demonstrates also by drawings from specimens, that the capacity of the pelvis may be altered or interfered with, by the existence of the affection, by which he controverts the assertion of Dupuytren, that the pelvis is not affected by the occurrence of congenital dislocations of the femur. He does not agree with others that such cases are most likely to occur when one side only is affected. He admits, however, that after middle life, the conformation of the head of the femur and that of the acetabulum and articular capsule become so altered as to render the case hopeless, and only limits a favorable prognosis to such cases as fall into the hands of the surgeon in early life, or as he states in the next chapter, the patient must not have passed beyond the twelfth or fourteenth year of age.

Chapter 7th. The pathology is shown to vary very much, according to the extent and duration of the displacement; yet there are certain post-mortem appearances which belong to the affection under all circumstances. In early life, the head of the femur, the acetabulum, its cartilages, and its ligaments, may have undergone very little actual change, but "in proportion to the duration of the dislocation, and to the more or less advanced period of life, the nutrition of the parts from various causes being materially disturbed, the structural changes in the various tissues become also more marked."

In the sequel, our author points out these changes very clearly. Those occurring in the cotyloid cavity being not only a speedy alteration in its size and shape, but also a deposition occasionally on its surface, with an alteration of its investing cartilage. The head of the femur soon alters in its shape, dimensions, and texture, too much so, we fear, to justify even so much hope as our author entertains of the success of any plan of treatment which may be attempted, especially in those cases in which "the nutrition of the head may become so perverted as to produce structural deterioration, terminating sometimes in its total disappearance."

The capsule and ligamentum teres may remain for a considerable time unaltered, except in being much elongated, but ultimately the former will become much attenuated, and in consequence of the constant pressure of the head of the femur, and the friction on the dorsum of the ilium, will become perforated so as to allow the bone to escape from its investment, while the distended ligamentum teres will rupture, and afford to the joint no further support; or they may coalesce, forming a strong ligamentous cord, uniting the upper portion of the femur to the pelvis. These conditions, together with the formation of a new capsule which sometimes takes place, are well shown in plates 6, 7, 8, and 9.

Our author believes that a new socket or acetabulum may be formed on the dorsum of the ilium, after the head of the femur has reached that location, in two different ways: 1st. At the expense of the ilium, out of which a shallow cavity is scooped by its continued pressure; and 2d. By a deposition of new osseous matter. M. Guérin believes that the latter mode of formation of a new socket always occurs, from which opinion our author disagrees, and in plate No. 7 shows that the simple depression on the dorsum of the ilium for the lodgment of the head of the bone does sometimes take place.

The altered appearance of the muscles about the articulation, the atrophied state of some of them, and the contracted condition of others, while others are changed in their texture, are well described.

The changes also in the vessels, nerves, cellular tissue and skin about the parts, as well as the alterations in the osseous tissues, are sufficiently dwelt upon. Our author also shows by actual measurement of morbid specimens, that the pelvis becomes altered in its configuration and capacity, and points out the difficulties which may result during parturition, from the existence of the affection under consideration.

A clear analysis of malformations of the pelvis, &c., and of the causes and methods of operation in their production, is given, and much influence is very properly ascribed to the faulty direction of muscular contraction, aided by the pressure of the trunk on the pelvis, the bony structure of which is perhaps frequently diseased.

In confirmation of his views of the pathology of congenital luxation of the head of the femur on the dorsum of the ilium, our author details minutely the post mortem appearances presented on dissection of two subjects, one female and the other male, which embrace almost every thing which he previously states to exist, including the many and extensive alterations in the different soft and hard structures about the thighs and hips. In the case of the male, the extent of the deformity and shortening of the femurs themselves, must have been such as to have rendered locomotion extremely imperfect.

Chapt. 8th. *Treatment.*—Our author fully appreciates the difficulties to be encountered in the treatment of the deformity, and it could scarcely be otherwise, when the extensive alterations which he has shown to occur in most cases, are taken into consideration. The practitioner undertakes with diffidence the reduction of a common traumatic luxation (no matter of what bone) when more than a few weeks have elapsed since its occurrence, because he is aware of the changes which invariably take place to a greater or less extent, not only in the structures belonging to the joint itself, but also in those in its neighborhood; how much more distrustful then must he be of his efforts for the relief of an affection which dates its existence from the birth, and the difficulties of which have grown with the growth of its unfortunate victim, more especially when a number of years have probably passed before his assistance is demanded.

Dupuytren, with all his pathological knowledge, and full of surgical skill as he was, deemed such cases hopeless, and rested content with a palliative means of relief, in the form of strong plates so adjusted and fixed as to retain to a certain extent the heads of the bones in their unnatural positions, and to prevent them from sliding up and down at every effort of locomotion. But as one fact of right takes precedence of all hypotheses, no matter how plausible, so it appears that cases of luxation of the head of one or of both femurs have been successfully treated, and consequently attempts at their reduction may hereafter be undertaken with a prospect of a fortunate result. Unsuccessful attempts for the relief of the affection were first made by MM. Duval and Lafond, and subsequently by MM. Humbert and Jacquier, which, however, directed the attention of the profession to its management. By the report of a commission of the Royal Academy of Medicine of Paris, made in 1838, it appears that M. Pravaz succeeded in the reduction of a congenital luxation of the head of the femur upon the ilium. Other cases detailed by M. Pravaz, as well as those given by other practitioners, demonstrate the occasional curability of the deformity. "In 1843 the council general of the civil hospitals of Paris nominated a commission," which reported the successful termination of three cases of the deformity treated by M. Guérin at the *Hôpital des Enfans*, and M. Guérin has himself reported other successful results.

As the disease may be said to be chronic, so is the treatment "necessarily prolonged and tedious," and can only be justified by the prospect of ultimate relief to a deformity not less distressing than the most horrible of its class.

The treatment should be commenced as soon as possible, and it has proved successful from the age of three to that of fifteen years.

The younger the patient, the fewer and less permanent are the changes in the structures about the parts.

After remarking on the condition of things which may justify the operation, our author divides the curative treatment into three periods, viz.: "First, the preparatory extension; secondly, the reduction; thirdly, the normal consolidation of the articular structures, so that the head of the femur may be permanently retained in the acetabulum." The preparatory extension having for its object the dislodgment of the head of the femur from its unnatural position on the dorsum of the ilium, and the stretching of the contracted muscles and fascia, must be gradual, and should be kept up for a long time, varying from two to six months. For this purpose the complicated apparatus devised by M. Pravaz is recommended, and of it a plate is given.

When enough extension has been obtained, the reduction is to be attempted by abducting the limb, and by pushing the head of the femur from above downwards and from without inwards. Sometimes the reduction is immediate and complete, at others "the head of the femur can only be brought into its anatomical position and retained there," when it should be fixed by some appropriate contrivance until nature has effected such changes in the acetabulum as to render it fit for its permanent lodgment.

After the articulation has been established, and when the muscles, &c., have to a certain extent assumed their normal condition, the third period of the treatment may be commenced, which consists in the use of an apparatus in which the limbs can be exercised, while the weight of the body is not allowed to rest upon them, until the parts have become fully consolidated. Subsequently crutches are recommended at the convenience of the patient. Appropriate apparatus for fulfilling the above indications are described, and well executed plates given in illustration.

The plans recommended and practised by M. Guérin to assist in the reduction and retention of the head of the femur, do not seem to find much favor in the eyes of our author, although he says nothing against their employment. They consist of a subcutaneous division of the resisting muscles and fascia, the making in the same way an opening in the closed capsule to allow the head of the bone to return, and of scarifications about the acetabulum, so as to excite sufficient inflammation to cause a deposition to be thrown out which will retain the head of the femur in its new though natural position.

To Dr. Carnachan we take pleasure in returning our thanks for presenting to the profession so interesting a treatise, which will doubtless fall into the hands of surgeons generally, by whom it will be duly appreciated; and to the publishers our thanks are due for the handsome style in which the work appears.

We trust that at some future day the author will favor us with his views on other important deformities of the bones and joints.

